# Current Distribution of Selected Vector-Borne Diseases in Dogs in Spain

**DOI:** 10.3389/fvets.2020.564429

**Published:** 2020-10-22

**Authors:** José Alberto Montoya-Alonso, Rodrigo Morchón, Noelia Costa-Rodríguez, Jorge Isidoro Matos, Yaiza Falcón-Cordón, Elena Carretón

**Affiliations:** ^1^Internal Medicine, Faculty of Veterinary Medicine, Research Institute of Biomedical and Health Sciences (IUIBS), University of Las Palmas de Gran Canaria, Las Palmas de Gran Canaria, Spain; ^2^Animal and Human Dirofilariosis Group, Laboratory of Parasitology, Faculty of Pharmacy, University of Salamanca, Salamanca, Spain

**Keywords:** *Dirofilaria immitis*, *Leishmania infantum*, *Anaplasma* spp., *Ehrlichia canis*, epidemiology, seroepidemiology, vector-borne diseases, zoonosis

## Abstract

Currently, climate change, modifications of landscapes and habitats due to human activities, as well as an increase in the movement of reservoirs and new species of competent vectors, have contributed to the spread of canine vector-borne diseases. These are mostly emerging and neglected diseases, some of them with zoonotic potential. Therefore, the objective of this study was to assess the prevalence and distribution of four major canine vector-borne diseases (*Dirofilaria immitis, Leishmania infantum, Anaplasma* spp., and *Ehrlichia canis*) in Spain. Between September 2018 and February 2020, blood was sampled from 4643 client-owned dogs from 111 veterinary clinics from the 17 autonomous communities of Spain. All samples were tested for the detection of *D. immitis* antigens, and for antibodies against *L. infantum, Anaplasma* spp. and *E. canis*. Of the studied dogs, 22.14% were positive for one or several diseases while the prevalence was 6.25% (CI: 5.59–6.98) for *D. immitis*, and the seroprevalences were 10.36% (CI: 9.52–11.27) for *L. infantum*, 5.06% (CI: 4.47–5.73) for *Anaplasma* spp., and 4.26% (CI: 3.72–4.88) for *E. canis*. Co-infections by two and three vector-borne diseases were reported in 13% and 2% of the infected dogs, respectively. The studied vector-borne diseases are widely distributed throughout the Spanish geography, being observed and expanding northward in the case of *D. immitis* and *L. infantum*. The results point to an insufficiency of preventive measures to avoid the infection, and the need of the implementation of awareness campaigns among veterinarians and owners. Furthermore, a close collaboration between veterinarians, physicians and health authorities would be necessary for such zoonotic vector-borne diseases.

## Introduction

It is described that climatic factors (i.e., temperature and humidity) influence the distribution of animal diseases, such as vector-borne diseases, causing dynamic changes in their geographical distribution, epidemiology, pathogenicity, and control. In addition, anthropogenic factors such as globalization with the increasing circulation of people and goods, the transport of infected animals from endemic areas, environmental changes related to human activities and climatic change, favor the spread of vector-borne diseases (1–3).

Canine vector-borne diseases include a variety of diseases which are mainly transmitted by culicid mosquitoes, phlebotomine sand flies, fleas, and ticks. This research focuses on four canine vector-borne diseases that are among the most clinically relevant and most prevalent in the Spanish geography (1, 3, 4).

Canine dirofilariosis, caused by *Dirofilaria immitis*, is a chronic and potentially lethal disease transmitted by culicid mosquitoes (5). In Spain, prediction models based on geo-environmental features and epidemiological data establish that the highest risk and prevalence occurs in the Canary and Balearic Islands, the southern and eastern peninsular coastal areas and within peninsular areas with irrigated crops and wetlands (1, 6–8). Canine leishmaniasis is caused by the protozoan *Leishmania infantum*, and transmitted by the bite of *Phlebotomus* spp.; the infection can cause a severe clinical presentation which can lead to death or serious systemic repercussions (9–12). In Spain, canine leishmaniasis was considered to be limited to the Mediterranean region (13, 14) but currently is considered endemic in most of the Iberian Peninsula and the Balearic Islands with prevalences between 3.7 and 46.6%, being higher in southern and eastern areas of the country (4, 14–18). Both dirofilariosis and leishmaniasis show long asymptomatic periods, which contribute to their spread. Furthermore, lack of preventative measurements and awareness in non-endemic areas may also be contributing to expansion.

*Anaplasma* spp. and *Ehrlichia canis* are intracellular gram-negative bacteria, causative agents of infectious cyclic thrombocytopenia (*A. platys*), granulocytic anaplasmosis (*A. phagocytophilum*), and monocytic ehrlichiosis (*E. canis*). These mostly affect dogs and are mainly transmitted by *Rhipicephalus sanguineus* and *Ixodes ricinus* (19, 20). They cause a disease mainly characterized by mild to severe thrombocytopenia causing bleedings, lethargy, anorexia, fever, lymphadenomegaly, or splenomegaly, in addition to other clinical signs (3, 21, 22). In Europe, their presence has been reported (3), but in Spain there are few studies reporting these diseases being widely distributed in the northeast, center and southwest of the country, with prevalences between 1 and 20% (9, 23).

*Ehrlichia canis* is not considered an important zoonotic agent, although some clinical cases have been reported (24). However, *Anaplasma* spp*., D. immitis* and *L. infantum* have an important zoonotic potential (5, 9, 25–27). In humans, *D. immitis* produces parasitic granulomas in the pulmonary parenchyma, which can be confused with lung cancer (5); *L. infantum* can present two clinical forms: visceral and cutaneous. The cutaneous form can resolve on its own with the formation of scars. However, visceral leishmaniasis is an important zoonotic disease in southern Europe, which can be fatal especially in immunocompromised patients (28). *Anaplasma* spp. can cause clinical manifestations ranging from mild self-limiting febrile illness, to fatal infections (26).

In Spain, the distribution of these vector borne-diseases is incomplete because epidemiological studies have not been carried out in some provinces and autonomous communities. Moreover, some of these studies were done more than 20 years ago, and in view of the quick spread of vector-borne diseases described in other European countries (29–31), new data should be published to solve this lack of data and present an epidemiological map of their possible expansion that shows a better knowledge of these diseases and allows a more effective fight against them. Therefore, due to the importance and spread of these vector-borne diseases, the objective of this study was to update and complete the epidemiological map of canine *D. immitis, L. infantum, Anaplasma* spp., and *E. canis* in Spain, estimating prevalences in the Spanish autonomous communities.

## Materials and Methods

### Climatic Characteristics of the Study Area

The Köppen Climate Classification system was applied to identify the different types of climate within Spain (32, 33). According to this, temperate climates are the predominant types in the country.

The temperate with dry or hot summer climate (Csa) is the type of climate which covers most of the Iberian Peninsula and the Balearics, occupying ~40% of its surface. This climate is mainly found in Catalonia, Valencian Community, the Balearic Islands, Andalusia, Castilla La Mancha, Madrid, and Extremadura. This climate is characterized by having hot summers with the average temperature in the warmest month >22°C. The temperate with dry or temperate summer climate (Csb) is the second most common climate in Spain, occurring in ~22% of the territory and is mainly present in Galicia and Castile and León. Like the Csa climate, it has a minimum of rainfall in the summer, but the summer is mild as it does not exceed 22°C on average in the warmest month. The third most common climate in Spain is the temperate with a dry season and temperate summer climate (Cfb). It is located in the northern region and mainly presented in Asturias, Cantabria, Basque Country, La Rioja, Navarra, and Aragón. Murcia is mainly represented by the cold steppe climate (Bsk); this correspond to a dry climate which is characterized as such because the evaporation exceeds precipitation on average, but is less than potential evaporation; average temperature is <18°C. Finally, the Canary Islands present different climates but, overall, the climate is subtropical and desert, moderated by the sea and in summer by the trade winds (Subtr).

### Sample Collection and Analysis

**Figure 1:**
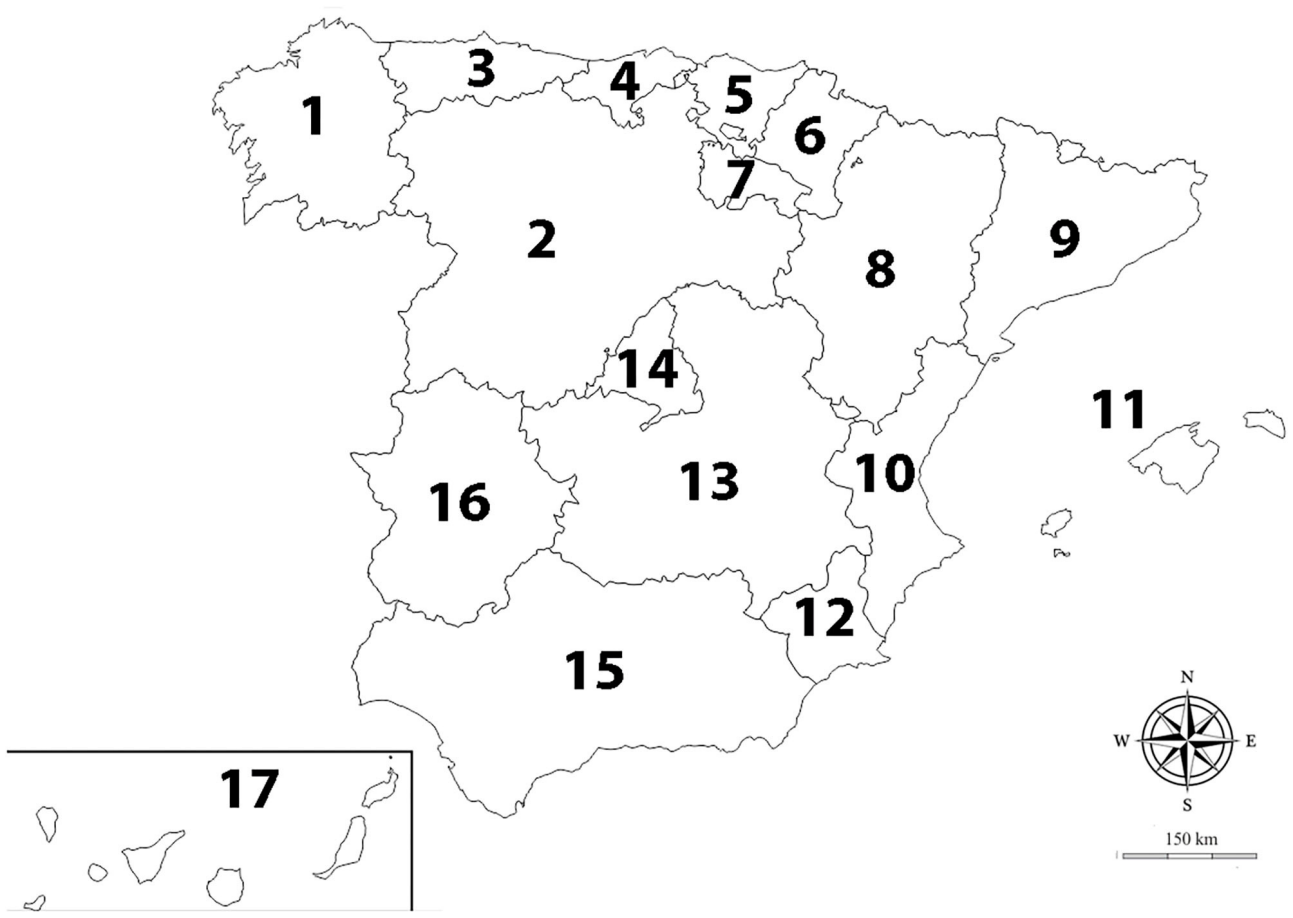
Autonomous communities of Spain. (1) Galicia; (2) Castile and Leon; (3) Asturias; (4) Cantabria; (5) Basque Country; (6) Navarra; (7) La Rioja; (8) Aragon; (9) Catalonia; (10) Valencian Community; (11) Balearic Islands; (12) Murcia; (13) Castilla-La Mancha; (14) Madrid; (15) Andalusia; (16) Extremadura; (17) Canary Islands.

The study included a total of 4,643 blood samples from domestic dogs, collected between September 2018 and February 2020. Samples were collected from dogs presented for routine health examination to 111 veterinary clinics and hospitals located in the 17 autonomous communities present in Spain (Figure 1 and Table 1); practices were asked to choose the samples at 'random'. The participation of clinics and hospitals was voluntary and samples were collected during the period of time in which the study lasted. The criteria for inclusion were no having previous history of infection by the studied vector-borne diseases, not receiving regular chemoprophylaxis for the studied vector-borne diseases, and owner consent to participate in the survey. Epidemiological data, such as gender, age at presentation to the clinics and habitat (where the dog spends all or most of its time: indoor, outdoor, or both), were recorded.

**Table 1:** Prevalence and seroprevalences of the studied vector-borne diseases in each autonomous community in Spain.

**Regions**		**Climate**	**Veterinary centers**	***n***	**+ *D. immitis***	**%**	**+ *L. infantum***	**%**	**+ *Anasplama* spp**.	**%**	**+ *E. canis***	**%**
1	Galicia	Csb	6	330	21	6.36	15	4.55	10	3.03	8	2.42
2	Castile and Leon	Csb	8	401	25	6.23	23	5.74	11	2.74	8	2.00
3	Asturias	Cfb	3	116	1	0.86	1	0.86	2	1.72	5	4.31
4	Cantabria	Cfb	4	147	2	1.36	3	2.04	7	4.76	6	4.08
5	Basque Country	Cfb	6	104	0	0.00	2	1.92	3	2.88	5	4.81
6	Navarra	Cfb	3	107	2	1.87	7	6.54	3	2.80	6	5.61
7	La Rioja	Cfb	4	121	8	6.61	8	6.61	3	2.48	5	4.13
8	Aragon	Cfb	6	131	10	7.63	21	16.03	15	11.45	9	6.87
9	Catalonia	Csa	10	510	39	7.65	69	13.53	16	3.14	21	4.12
10	Valencian Community	Csa	5	374	26	6.95	64	17.11	26	6.95	34	9.09
11	Balearic Islands	Csa	8	180	11	6.11	38	21.11	9	5.00	6	3.33
12	Murcia	Bsk	2	223	18	8.07	55	24.66	22	9.87	11	4.93
13	Castilla-La Mancha	Csa	8	237	9	3.80	17	7.17	9	3.80	12	5.06
14	Madrid	Csa	10	461	15	3.25	38	8.24	16	3.47	21	4.56
15	Andalusia	Csa	11	427	29	6.79	77	18.03	38	8.90	25	5.85
16	Extremadura	Csa	5	202	17	8.42	29	14.36	16	7.92	5	2.48
17	Canary Islands	Subtr	12	572	57	9.97	14	2.45	29	5.07	11	1.92
	Total		111	4,643	290	6.25	481	10.36	235	5.06	198	4.26

*Veterinary centers: number of veterinary clinics and hospital participating in the study. n, number of dogs sampled; +, positive animals; %, percentage of positive animals; Csa, temperate with dry or hot summer climate; Csb, temperate with dry or temperate summer climate; Cfb, temperate with a dry season and temperate summer climate; Bsk, cold steppe climate; Subtr, subtropical*.

Blood samples were collected from the cephalic or jugular vein, placed in 3 ml serum tubes and centrifuged. Serum samples were kept at −20°C until tests were performed. All samples were tested for the detection of *D. immitis* antigens and for the detection of antibodies against *L. infantum, E. canis*, and *Anaplasma* spp. following immunochromatography techniques by using Uranotest Quattro (Uranovet, Barcelona Spain) following manufacturer's instructions. Briefly, one drop of serum or plasma was added along with two drops of reagent to each of the test strips. The sensitivity of the tests was: 97% for *L. infantum* (vs. IFI), 95% for *Ehrlichia* (vs. IFI), 94% for *D. immitis* (vs. necropsy) and 96% for *Anaplasma* (vs. IFI). The specificity was: 99% for *L. infantum* (vs. IFI), 94.6% for *Ehrlichia* (vs. IFI), 100% for *D. immitis* (vs. necropsy), and 99% for *Anaplasma* (vs. IFI).

### Statistical Analysis

Data were analyzed using SPSS Base 20.0 software (SPSS Inc./IBM, Chicago, Illinois, USA). Descriptive analysis of the qualitative variables was carried out considering the number of cases and percentages. Univariate and multivariate logistic regression models were carried out to establish the degree of association between the variables gender, age, presence of co-infection, climate, habitat, region and the presence of *Dirofilaria, Leishmania, Anaplasma*, and *Ehrlichia*. Each variable was analyzed individually by obtaining crude odds ratios (ORs) and their 95% confidence intervals. Subsequently, the OR values adjusted for the rest of the factors, using logistic regressions, and their corresponding 95% confidence intervals were estimated as well.

## Results

Of the studied dogs, 54.3% (2,521/4,643) were male and 45.7% (2,122/4,643) were female. The age ranged from 2 months to 19 years old, and dogs were further divided into four age groups: dogs <1 year (4.6%; 214/4,643), from 1 to 5 years (36.1%; 1676/4,643), from 5 to 10 years (37.6%; 1,742/4,643), from 10 to 15 years (19.9%; 926/4,643), and dogs >15 years (1.8%; 85/4,643). Regarding habitat, 30% (1,393/4,643) of the dogs were indoor (dogs always kept inside the house), 37.2% (1,726/4,643) were outdoor (those always kept outside the house) and 32.8% (1,524/4,643) for dogs that spent at least 1–50% of their time outdoors (indoor/outdoor).

**Figure 2:**
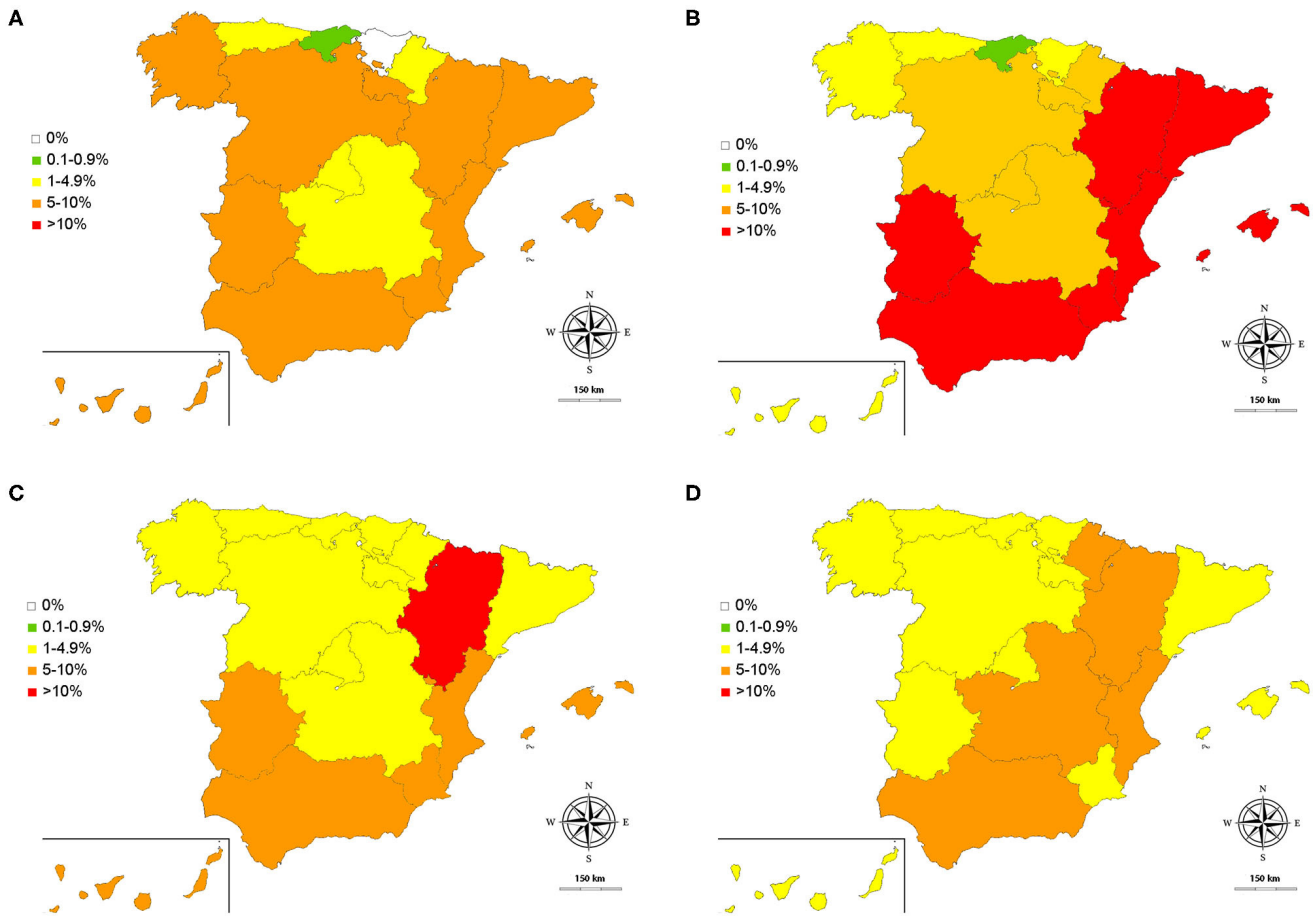
Obtained prevalences for *D. immitis* **(A)**, and seroprevalences for *L. infantum* **(B)**, *A. platys* **(C)**, and *E. canis* **(D)** in Spain: 0% (◻); 0.1–0.9% (◼); 1–4.9% (◼); 5–10% (◼); >10 (◼).

The results showed that 22.14% (1,028/4,643) were positive for one or several diseases. The overall prevalence of *D. immitis* was 6.25% (290/4,643) (95% CI: 5.59–6.98) and the seroprevalences of *L. infantum, Anaplasma* spp., and *E. canis* were 10.36% (481/4,643) (95% CI: 9.52–11.27), 5.06% (235/4,643) (95% CI: 4.47–5.73) and 4.26% (198/4,643) (95% CI: 3.72–4.88), respectively. Results by autonomous communities are shown in Figure 2 and Table 1, while results by gender, groups of age and habitat are shown in Table 2.

**Table 2:** Prevalence and seroprevalences of vector-borne diseases studied by gender, age, and habitat.

	***n***	**%**	**+ *D. immitis***	**%**	**CI (95%)**	**+ *L. infantum***	**%**	**CI (95%)**	**+ *Anaplasma* spp**.	**%**	**CI (95%)**	**+ *E. canis***	**%**	**CI (95%)**
**Gender**														
Male	2,521	54.3	138	5.47	(4.65, 6.43)	271	10.75	(9.60, 12.02)	129	5.12	(4.32, 6.05)	97	3.85	(3.16, 4.67)
Female	2,122	45.7	152	7.16	(6.14, 8.34)	210	9.90	(8.70, 11.24)	106	4.99	(4.15, 6.01)	101	4.76	(3.93, 5.75)
**Age**														
<1 year	214	4.6	5	2.34	(1.00, 5.35)	14	6.54	(3.94, 10.68)	6	2.80	(1.29, 5.98)	2	0.93	(0.26, 3.34)
1–5 years	1,676	36.1	107	6.38	(5.31, 7.66)	179	10.68	(9.29, 12.25)	75	4.47	(3.58, 5.57)	66	3.94	(3.11, 4.98)
5–10 years	1,742	37.6	114	6.54	(5.48, 7.80)	194	11.14	(9.74, 12.70)	80	4.59	(3.71, 5.68)	73	4.19	(3.35, 5.24)
10–15 years	926	19.9	60	6.48	(5.07, 8.25)	87	9.39	(7.68, 11.45)	73	7.88	(6.32, 9.80)	45	4.86	(3.65, 6.44)
>15 years	85	1.8	4	4.71	(1.85, 11.48)	7	8.23	(4.05, 16.04)	1	1.18	(0.21, 6.37)	5	5.88	(2.54, 13.04)
**Habitat**														
Indoor	1,393	30	25	1.79	(1.22, 2.64)	59	4.23	(3.30, 5.42)	14	1.00	(0.60, 1.68)	10	0.72	(0.39, 1.32)
Outdoor	1,726	37.2	158	9.15	(7.88, 10.61)	269	15.59	(13.95, 17.37)	130	7.53	(6.38, 8.87)	92	5.33	(4.37, 6.49)
Indoor/Outdoor	1,524	32.8	107	7.02	(5.84, 8.41)	153	9.91	(8.63, 11.65)	91	5.97	(4.89, 7.28)	96	6.30	(5.19, 7.63)
Total	4,643		290	6.25	(5.59, 6.98)	481	10.36	(9.52, 11.27)	235	5.06	(4.47, 5.73)	198	4.26	(3.72, 4.88)

*n, number of dogs sampled; +, positive animals; %, percentage of positive animals*.

Taking into account the sensitivity and specificity of the tests used, true prevalence could be estimated as 6.65% (*D. immitis*), 10.78% (*L. infantum*), 5.32% (*Anaplasma* spp.), and 4.69% (*E. canis*).

Co-infections were observed in 15% of the infected dogs. Of them, 13% were infected by two vector-borne infections, being *L. infantum* + *E. canis* (n=41), *L. infantum* + *Anaplasma* spp. (*n* = 32), *L. infantum* + *D. immitis* (*n* = 19), *D. immitis* + *Anaplasma* spp. (*n* = 19), *E. canis* + *Anaplasma* spp. (*n* = 17), *D. immitis* + *E. canis* (*n* = 6). Furthermore, 2% of them were co-infected by three vector-borne infections (*E. canis* + *L. infantum* + *Anaplasma* spp., *n* = 13; *D. immitis* + *E. canis* + *L. infantum, n* = 8).

By climates, *D. immitis* and *L. infantum* trended to show lower prevalences in the autonomous communities dominated by the Cfb climate; while higher prevalences were observed in the Canary Islands (*D. immitis*), and Csa and Bsk climates (*L. infantum*). *Anaplasma* spp. and *E. canis* were quite homogeneously distributed in the country.

Gender and age were significantly associated with the presence of *D. immitis*, being females 43% more likely to present dirofilariosis. No significant association was observed between gender and the presence of *Leishmania, Anaplasma* and *Ehrlichia* (Table 3).

**Table 3:** Factors associated with the presence of *D. immitis, L. infantum, Anaplasma* spp., and *E. canis*.

**Variables**	***D. immitis***		***L. infantum***		***Anaplasma*** **spp**.		***E. canis***	
	**Crude ORs (CI 95%)**	**Adjusted ORs (IC 95%)**	**Crude ORs (CI 95%)**	**Adjusted ORs (IC 95%)**	**Crude ORs (CI 95%)**	**Adjusted ORs (IC 95%)**	**Crude ORs (CI 95%)**	**Adjusted ORs (IC 95%)**
**Gender**^**#**^	1.33 (1.05–1.69)*	1.43 (1.12–1.82)*	1.43 (1.12–1.82)	0.96 (0.79–1.18)	0.97 (0.74–1.26)	0.99 (0.75–1.31)	1.24 (0.93–1.66)	1.33 (0.99–1.78)
**Age**								
<1 year	Ref.	Ref.	Ref.	Ref.	Ref.	Ref.	Ref.	Ref.
1–5 years	2.85 (1.14–7.07)*	3.03 (1.21–7.59)*	1.70 (0.97–3.00)	2.07 (1.16–3.69)*	1.62 (0.69–3.77)	1.47 (0.62–3.46)	2.97 (0.92–9.53)	3.05 (0.94–9.86)
5–10 years	2.92 (1.18–7.25)*	3.38 (1.35–8.43)*	1.79 (1.02–3.14)*	2.27 (1.28–4.05)*	1.66 (0.71–3.87)	1.67 (0.71–3.93)	3.16 (0.98–10.12)	3.37 (1.04–10.87)*
10–15 years	2.89 (1.14–7.30)*	3.10 (1.21–7.88)*	1.48 (0.82–2.65)	1.62 (0.88–2.95)	2.92 (1.25–6.81)*	2.93 (1.24–6.93)*	3.76 (1.15–12.19)*	3.86 (1.17–12.66)*
>15 years	2.06 (0.541–7.8)	2.21 (0.57–8.57)	1.28 (0.49–3.29)	1.09 (0.41–2.91)	0.83 (0.16–4.22)	0.58 (0.11–3.05)	4.39 (1.02–18.82)*	4.21 (0.97–18.24)
**Co–infection**								
*D. immitis*	–	–	1.60 (1.14–2.24)*	1.26 (0.88–1.79)	2.23 (1.48–3.36)^˝^	1.57 (1.01–2.41)*	1.53 (0.93–2.52)	1.14 (0.68–1.91)
*L. infantum*	1.60 (1.14–2.24)*	1.25 (0.87–1.78)	–	–	2.63 (1.90–3.64)^˝^	1.82 (1.29–2.57)*	2.29 (1.60–3.29)^˝^	1.59 (1.09–2.31)*
*Anaplasma* spp.	2.23 (1.48–3.36)^˝^	1.64 (1.07–2.50)*	2.63 (1.90–3.64)^˝^	1.79 (1.26–2.53)*	–	–	3.85 (2.56–5.80)^˝^	2.65 (1.73–4.05)^˝^
*E. canis*	1.53 (0.93–2.52)	1.13 (0.67–1.89)	2.29 (1.60–3.29)^˝^	1.56 (1.07–2.29)*	3.85 (2.56–5.80)^˝^	2.62 (1.71–4.01)^˝^	–	–
**Climate**								
Bsk	Ref.	Ref.	Ref.	Ref.	Ref.	Ref.	Ref.	Ref.
Cfb	0.37 (0.19–0.70)*	0.34 (0.14–0.77)*	0.18 (0.12–0.29)^˝^	0.27 (0.15–0.48)^˝^	0.43 (0.24–0.76)*	1.91 (0.82–4.47)	1.00 (0.50–2.01)	1.62 (0.63–4.14)
Csa	0.74 (0.44–1.23)	0.91 (0.49–1.70)	0.49 (0.35–0.68)^˝^	0.74 (0.49–1.11)	0.52 (0.32–0.84)*	1.04 (0.59–1.85)	1.05 (0.56–1.98)	1.35 (0.64–2.84)
Csb	0.76 (0.43–1.34)	0.75 (0.35–1.64)	0.16 (0.10–0.26)^˝^	0.25 (0.14–0.45)^˝^	0.27 (0.14–0.50)^˝^	1.12 (0.46–2.73)	0.43 (0.19–0.94)*	0.80 (0.29–2.20)
Subtr	1.26 (0.72–2.19)	1.54 (0.87–2.75)	0.07 (0.04–0.14)^˝^	0.08 (0.04–0.15)^˝^	0.48 (0.27–0.86)*	0.69 (0.37–1.26)	0.37 (0.16–0.88)*	0.47 (0.19–1.12)
**Habitat**								
Indoors	Ref.	Ref.	Ref.	Ref.	Ref.	Ref.	Ref.	Ref.
Indoors/Outdoors	4.13 (2.65–6.42)^˝^	4.14 (2.65–6.48)^˝^	2.52 (1.85–3.43)^˝^	2.43 (1.77–3.34)^˝^	6.25 (3.54–11.03)^˝^	5.44 (3.06–9.66)^˝^	0.12 (0.06–0.24)^˝^	8.28 (4.27–16.02)^˝^
Outdoors	5.51 (3.59–8.46)^˝^	5.48 (3.55–8.46)^˝^	4.17 (3.11–5.58)^˝^	3.76 (2.79–5.08)^˝^	8.02 (4.60–13.99)^˝^	6.76 (3.84–11.90)^˝^	1.19 (0.88–1.60)	6.67 (3.43–12.98)^˝^
**Regions**								
North	Ref.	Ref.	Ref.	Ref.	Ref.	Ref.	Ref.	Ref.
Centre	0.97 (0.72–1.31)	0.66 (0.44–1.00)	1.79 (1.42–2.24)^˝^	0.88 (0.65–1.20)	1.49 (1.07–2.08)*	1.91 (1.09–3.35)*	1.47 (1.06–2.03)*	1.35 (0.81–2.23)
South	1.60 (1.21–2.11)^˝^	0.83 (0.50–1.39)	1.65 (1.30–2.10)^˝^	1.35 (0.94–1.95)	2.12 (1.54–2.93)^˝^	3.32 (1.80–6.14)^˝^	1.03 (0.71–1.50)	1.38 (0.75–2.54)

OR, Odds Ratio; CI, confidence intervals. “North” included the autonomous communities: Asturias, Cantabria, Basque Country, Galicia, Navarra, La Rioja, Castile and Leon, Aragon, and Catalonia. “Center” included the autonomous communities: Madrid, Valencian Community, Extremadura, Castilla La Mancha, and Balearic Islands. “South” included the autonomous communities: Andalusia, Murcia and Canary Islands. Bsk, cold steppe climate; Cfb, temperate with a dry season and temperate summer climate; Csa, temperate with dry or hot summer climate; Csb, temperate with dry or temperate summer climate; Subtr, subtropical climate. *p < 0.05; ^˝^p < 0.001; ^#^Reference category = male.

Regarding age, a significant association was observed with the presence of *Leishmania*; as age increased, the risk of leishmaniasis increased, except in dogs >15 years, in which the association was no longer significant. In dogs infected by *Anaplasma* spp., only the group of 10–15 years presented a significant association, being the risk of presenting infection 2.93 times higher than in dogs <1 year. When dogs infected by *E. canis* were analyzed, those between 5 and 10 years showed 3.37 times more risk of presenting the infection, and those between 10 and 15 years showed 3.86 times more risk of infection, when compared to dogs <1 years, both associations being significant. The rest of the age groups studied did not show any significant association with the presence of *E. canis*.

The risk of infection by *D. immitis* was significantly higher in dogs >1 year; also, co-infection with *Anaplasma* spp. significantly increased the risk of being infected by *D. immitis* by 64%. The indoor/outdoor or outdoor habitat was significantly associated with the presence of dirofilariosis, while the Cfb climate compared to the Bsk climate reduced the presence of dirofilariosis by 66%.

Co-infections with *Anaplasma* spp. and *E. canis* significantly increased the risk of *L. leishmaniasis* by 79 and 56%, respectively. Indoor/outdoor or outdoor habitat was significantly associated with the presence of *L. infantum*. The indoor/outdoor habitat presented 2.43 times more risk of being infected, while the outdoor habitat presented 3.76 times more risk of infection. Regarding climate, it was observed that the Cfb, Csb, and subtropical climates reduced the presence of *L. infantum* when compared to the Bsk climate, being a reduction of 73, 75, and 92%, respectively.

The presence of co-infections significantly increased the risk of anaplasmosis; infection by *D. immitis* increased this risk by 57% and *L. infantum* by 82%. Furthermore, dogs infected by *E. canis* showed a risk of anaplasmosis 2.62 times higher. A significant association was also observed between the presence of *Anaplasma* spp. and the region studied. Animals in the central zone showed an increase of 91% in presenting the disease compared to those that lived in the northern zone. Those in the southern zone had 3.32 times more risk of infection than those who lived in the northern zone.

Co-infection with *L. infantum* significantly increases the risk of suffering ehrlichiosis by 59%, while dogs infected with *Anaplasma* spp. had 2.65 times more risk of being co-infected by *E. canis*. No significant association was observed between the climate and the presence of *Anaplasma* spp. and *E. canis*. There was also no significant association with the region studied and the presence of dirofilariosis, leishmaniasis, and ehrlichiosis.

Although there is a relationship between the different variables and the presence of filaria and anaplasma, respectively, we must be cautious in interpreting the results because the goodness of fit of the model is not sufficient good as indicated by the Hosmer and Lemeshow test with a *p* < 0.05.

## Discussion

This study updates the epidemiology of *D. immitis, L. infantum, E. canis*, and *Anaplasma* spp. in the Spanish geography, completing the lack of data in some autonomous communities and updating the prevalences reported in others years ago.

When the results obtained for dirofilariosis were evaluated, it was observed that the prevalences obtained in this study maintain similar values with respect to the areas that had been evaluated in the last 5–6 years, such as Barcelona, Madrid, Salamanca, or the Canary Islands (7, 8, 34, 35). However, regarding studies carried out more than 25 years ago, two phenomena can be observed: on the one hand, an increase in the prevalence in endemic areas in the south and east [i.e., Extremadura, Murcia, Valencian Community, Catalonia, Aragon; (36–40)] and, on the other hand, progressive increase in the prevalence of areas with little presence of the parasite, or very localized presence, as is the case of Galicia (9, 36, 41), Castilla y León (36, 42), Castilla la Mancha (36, 43), or the Balearic Islands (23). Furthermore, prevalences of the parasite are reported for the first time in Asturias, Cantabria and Navarra. Only the Basque Country remains apparently free of dirofilariosis. Interestingly, a recently published work reports absence of canine dirofilariosis in northern Spain except in Navarra; although the tests used were of similar sensitivity and specificity, this difference is possibly due to the sampling method and number of samples (44). In any case, these results obtained in different studies should be interpreted with caution, due to the methodology used—sampling method, sample size and/or sensitivity, and specificity of the diagnostic test—in each of them. However, this shows the inexorable expansion of *D. immitis* throughout the Spanish geography as suspected, and as has already been reported at the European level (1). Curiously, there is an acute decrease in La Rioja of unknown causes (45). In general, the lowest prevalences corresponded to those autonomous communities dominated by the Cfb climate, probably due to the low temperatures that are present much of the year. These prevalences rise in La Rioja and Aragón, possibly because, despite the weather, they present important irrigated areas crossed by one of the main rivers of the Iberian Peninsula, the Ebro River.

The seropositivity toward *L. infantum* was the highest of all the vector-borne diseases analyzed and antibodies against *L. infantum* were detected in all the autonomous communities. The highest prevalences corresponded to the communities dominated by the Csa climate, especially those located in the east (Valencian Community, Murcia), southern Spain (Andalucía, Extremadura), and the Balearic Islands, which have been traditionally considered the endemic areas of the disease in Spain (9, 23, 46). Here, taking as reference studies of several areas or provinces (9, 13, 46–49), the prevalences remain high; a possible explanation being that the prophylactic measures are still insufficient. Likewise, as has been discussed previously, the different techniques and sampling carried out could be a confounding factor when comparing the variations in the prevalences obtained in the studies obtained in different years. The presence of *L. infantum* is confirmed throughout the north of the country, dominated by the Cfb climate, with the lowest seroprevalences in the entire country. Previously, except in the case of Galicia (9, 14) studies had been carried out in specific areas, such as towns or small areas of Cantabria, Asturias and the Basque Country, which in some cases demonstrated that leishmaniasis was already present (16), while a recent study also supports its presence in some autonomous communities in the north of the peninsula (44). The results of this research confirm not only the presence of leishmaniasis in the north, but its wide expansion throughout all the autonomous communities traditionally considered free of the disease, and, for the first time confirm its presence in some autonomous communities, such as La Rioja. In this study, the epidemiological map of the disease is completed, only studied in some provinces of the autonomous communities (i.e., Andalusia, Extremadura, Castile and Leon, Castilla la Mancha, Valencian Community, Catalonia, or Aragon) (9, 15, 17, 50–53). Furthermore, for the first time, the seroprevalence of *L. infantum* in the Canary Islands is estimated. These islands were considered free of the disease, although personal communications and a publication of a clinical case (54) raised suspicion of the presence of indigenous cases.

Both *Anaplasma* spp. and *E. canis* are distributed throughout the geography in a fairly homogeneous way. The presence of *Anaplasma* spp. may refer to *A. platys* or *A. phagocytophilum*. Although so far the latter has not been isolated in Spanish dogs, the main vector (i.e., *Ixodes ricinus*) is present in Spain (55), PCR-positive ticks have been found (56, 57) and this agent has also been isolated from other hosts, including humans (58–60). The presence of *Anaplasma* spp. and *E. canis* is quite homogeneous, without appearing to be influenced by the type of climate prevailing in the autonomous communities. This could be due to the fact that the vectors, *R. sanguineus* and *I. ricinus*, are widely distributed throughout the Spanish geography (61). Regarding *R. sanguineus*, this tick is the most common tick found in dogs in Spain (61–63) and it has been detected across the country, which could justify the homogeneous distribution found in this study (61, 64). The prevalences obtained from studies previously carried out in Galicia, Castilla, and Leon or Madrid on both bacteria (9, 65–68) were lower than the results obtained in this study, except in the case of Castilla and Leon (19.2% for *E. canis*) (66). However, before considering an increase or decrease in the prevalence in these autonomous communities, the methodology used and the type of sampling should be taken into account. In any case, this demonstrates the wide presence of *Anaplasma* spp. and *E. canis* in these regions. In addition, other previous studies have published seroprevalence data that were mostly reported in localized areas or provinces of the autonomous communities of Asturias, Aragon, Catalonia, Andalusia, the Valencian Community or the Balearic Islands (9, 23, 69); furthermore, a recent study reported presence of these diseases in the north of Spain, but showing lower prevalences, which could be due to the different sampling procedures—being the samples from the northernmost area of the studied autonomous communities—and number of samples (44). The results of this research have made it possible to complete and update the epidemiological map of these autonomous communities, as well as to provide prevalences in those communities that had never been epidemiologically evaluated.

Only significant differences were observed by gender in *Dirofilaria* infections; although other epidemiological studies found no significant differences (9, 14, 34), these differences were also observed in others (7, 8, 70). Regarding age, higher seropositivities were found in adult dogs between 1 and 10 years, observing a slight decrease in dogs >15 years. In all cases, the prevalences were lower in dogs <1 year, possibly due to less exposure to the pathogens and, in the case of *D. immitis*, due to the fact that antigens are not detected until 6 months post-infection. Previous epidemiological studies have obtained different results when the dogs were assessed by age, finding statistical significance between age groups in some epidemiological studies of vector-borne diseases but not in others (8, 9, 46). This could be due to the different methodology used in the studies, both in sampling and in the sensitivity/specificity of the different tests used.

By habitat, prevalences were significantly lower in dogs living indoors. This agrees with previous studies carried out in vector-borne diseases (9, 23, 34). This result is understandable since indoor animals are more protected against the presence of vectors. However, the results also show that this group of animals is at risk of infection, so prophylactic measures should be applied equally.

Co-infections were observed in 15% of the dogs; it has been previously reported that co-infections of vector-borne diseases are common, and studies have reported that up to half of all patients with positive test results for vector-borne diseases may have evidence of co-infection or co-exposure (71–74). Furthermore, the results showed that having some of the vector-borne infections studied increased the risk of showing other co-infections (i.e., *Dirofilaria* and *Anaplasma*; *Leishmania* and *Ehrlichia; Leishmania*, and *Anaplasma*). This highlights a lack of preventive measures to avoid the interaction with the vectors, and the need of the implementation of awareness campaigns among veterinarians and owners. The efficacy of the preventive measures against *D. immitis* has been observed in Gran Canaria with a constant decrease of the prevalences across 15 years (8, 70, 75).

In the case of *D. immitis*, the detection of parasitic antigens allows to determine current infections. However, the detection of antibodies that occur in the case of *Leishmania, Ehrlichia* and *Anaplasma* indicates exposure to disease-causing organisms, but the presence of antibodies does not always indicate disease, which, in some cases, may be confirmed by molecular techniques (3, 12). In any case, the presence of antibodies denotes that, whether or not it is a current infection, the animal is exposed to the disease and, therefore, at risk of infection, which is why preventive measures are necessary either way. On the other hand, absence of antibodies can also be found in infected animals confirmed by other techniques, such as PCR (76). Furthermore, some treated dogs might remain as reservoirs of vector-borne diseases. Therefore, even dogs that have been treated should be screened yearly for the presence of disease (77).

This study has a number of limitations. First, even though the samples were chosen randomly by veterinary clinicians, there is a possibility that they have inadvertently selected the samples out of some sort of personal preference. Likewise, it must be taken into account that the sample collected is not representative of each Autonomous Community, especially in those of great extension, despite the fact that efforts have been made to recruit the maximum number of veterinary clinics from different points of geography.

The results show a wide distribution of the vector-borne diseases studied; therefore, awareness of these diseases should be raised among the veterinary clinicians and dog owners, and prophylactic plans and measures should be implemented routinely. This is especially important in the vector-borne diseases studied in the present research, because all they are characterized by a wide range of symptoms, highlighting the presence of non-specific clinical signs or clinical-pathological anomalies that can lead to misdiagnosis. Also, many of these animals remain asymptomatic, being uncontrolled reservoirs of the infection; moreover, it has been described that animals with subclinical infections present an increased risk of disease transmission (78). In addition, we must consider the important zoonotic potential of the diseases studied, as the presence of human infections or risk of infection has already been described (5, 24, 25, 28). In this sense, it is essential to carry out a close collaboration between veterinarians, physicians and health authorities that allow, under the concept of One Health, to carry out a more rigorous and effective control of these diseases.

## Data Availability Statement

The raw data supporting the conclusions of this article will be made available by the authors, without undue reservation.

## Ethics Statement

This animal study was reviewed and approved by ethical committee of Veterinary Medicine Service of the University of Las Palmas de Gran Canaria. Written informed consent was obtained from the owners for the participation of their animals in this study.

## Author Contributions

EC, RM and JM-A designed the study and wrote the manuscript. RM, NC-R, JM, and YF-C performed the fieldwork and collected the data. All authors participated in the discussion of the results, corrected, and read and approved the final manuscript.

## Conflict of Interest

Although the article has been partially financed by Boehringer Ingelheim Spain, none of the authors maintains a contractual relationship with the company. The authors declare that the research was conducted in the absence of any commercial or financial relationships that could be construed as a potential conflict of interest.
